# Olive: A Potential Suppressor for Cervical Cancer by Upregulation of P21

**DOI:** 10.7759/cureus.38719

**Published:** 2023-05-08

**Authors:** Love Patel, Zuliang Deng, Ziwen Zhu, Marco Lequio, Jacob Hough, Huaping Xiao, Qian Bai, Mark R Wakefiel, Yujiang Fang

**Affiliations:** 1 Department of Immunology, Pathology, and Microbiology, Des Moines University, Des Moines, USA; 2 Pathology, University of Missouri, Columbia, USA; 3 School of Medicine, University of Missouri School of Medicine, Columbia, USA; 4 Biological Sciences, University of Missouri, Columbia, USA; 5 Urology, University of Missouri, Columbia, USA

**Keywords:** apoptosis, proliferation, p21, cervical cancer, olive extract

## Abstract

Background

Cervical cancer is the second deadliest for women between the ages of 20 and 39 years. Even with prevention tactics for screening, incident rates and mortality of cervical cancer remain high. Olive has been shown to have many beneficial effects in humans concerning cardiovascular disease and inflammation. Despite these promising benefits, little is known about its effect on cervical cancer. This study examined the effects and mechanism of effects of olive extract (OE) on the HeLa cervical cancer cell line.

Methodology

We utilized clonogenic survival assay, quick cell proliferation assay, and caspase-3 activity to investigate the effect of OE on the proliferation and apoptosis of the cervical cancer cell line HeLa. To investigate the mechanisms behind these findings, Reverse transcription polymerase chain reaction and immunohistochemistry were performed.

Results

OE inhibited the growth and proliferation of HeLa cells. In comparison to the control, the percentage of colonies, as well as the optical density of the cervical cancer cells, was found to be decreased. In addition, the relative activity of caspase-3, a marker for apoptosis, was increased after treatment with OE. The anti-proliferative effect of OE on HeLa cells correlated with the increase of an anti-proliferative molecule p21. However, the pro-apoptotic effect of OE was not correlated with the change in major pro-apoptotic or anti-apoptotic molecules examined in this study.

Conclusions

Our study suggests that OE inhibits the growth of HeLa cervical cancer cells by upregulation of p21. Further investigation of the effects of OE on cervical cancer and other cancers is warranted by these results.

## Introduction

Cancer is the second leading cause of death worldwide, just behind cardiovascular disease, and cervical cancer has the fourth highest incidence rate in women [1]. Although cervical cancer is a more preventable and treatable type of cancer, it continues to cause a significant amount of morbidity and mortality worldwide. In 2022, about 4,280 women are expected to die from cervical cancer in the United States [1]. While incidence and mortality rates of cervical cancer have been declining since the 1950s, there has been a concerning increase in the incidence of undetectable cervical cancer, specifically squamous cell carcinoma and adenocarcinoma, in recent years [2]. Almost all cervical cancers are due to the human papillomavirus (HPV) infection [3]. Reliable vaccines are available for HPV, but vaccination is far from being universal (57% coverage among female adolescents in the United States as of 2019) [4]. Until vaccination coverage and screening efforts improve, advances in the treatment of cervical cancer will be incredibly necessary. Early detection through cytology screening is the main mechanism by which cervical cancer is prevented. If cervical cancer develops, the primary treatment approach for progressed cancer involves a combination of surgery, radiation therapy, and chemotherapy [5], all of which are associated with a hefty financial cost and debilitating side effects.

One of the many components of naturally occurring olive extract (OE) is phenolic compounds, which have been shown to potentially improve cardiovascular health, contain anti-inflammatory properties, provide neuroprotection, and possess antimicrobial activity [6-9]. Other phytochemicals found in OE are oleuropein and flavonoids; these phytochemicals have been shown to be antioxidants and reduce cancer cell proliferation even at low molecular concentrations [10]. Few studies have discussed how other naturally occurring substances such as blueberries can be used therapeutically in cervical cancer, yet research on OE and cervical cancer is very limited [11-13]. This study was designed to demonstrate the effect of any proliferative and apoptotic properties of OE on a widely used human cervical cancer cell line HeLa.

This article was previously posted to the ResearchSquare preprint server on October 19th, 2022.

## Materials and methods

Tumor cell line

The American Type Culture Collection (Manassas, VA, USA) was the source of HeLa cells, the cervical cancer cell line. Dulbecco’s modified eagle medium with 10% heat-inactivated fetal bovine serum and 1% penicillin-streptomycin was used to maintain the cells and was provided by Invitrogen (Carlsbad, CA). The cultures were kept at 37°C in a humidified 5% CO_2_ incubator (Fisher Scientific, Pittsburgh, PA, USA). Once the HeLa cell cultures reached 70% confluence, they were subjected to designed experimental treatment regimens.

Treatment with OE

The concentration of OE and the duration of treatment were determined by our previous experiments [14]. We treated the 70% confluence HeLa cells with 50 µg/mL of OE for the treatment group, and the medium alone for the control group, both for 72 hours.

Clonogenic survival assay

The treatment was followed by a clonogenic survival assay; it was performed as outlined in previous experiments [14]. Results were expressed as a percentage of total colonies in the OE treatment group versus the control group.

Quick cell proliferation assay for measurement of HeLa cell proliferation

The activity of mitochondrial dehydrogenase directly correlates with the proliferation of viable cells; this produces formazan dye which was quantified by spectrophotometer. A quick cell proliferation assay kit from BioVision was employed, and a procedure that was detailed in our previous studies was followed closely [14].

Immunohistochemistry (IHC)

IHC staining p21 was done using a protocol described previously [14]. The dilutions used for primary (Santa Cruz Biotechnology Inc.) and secondary antibodies (Abs) were 1:200 and 1:500, respectively. To quantify p21+ cells, MetaMorph 6.3r6 image analysis software was used to measure the average staining intensity of proteins within the HeLa cell-covered zone. Results were expressed as the average integrated immunostaining intensity of three slides ±SEM, relative to the intensity of the control cells.

Reverse transcriptase-polymerase chain reaction (RT-PCR)

First, both the OE-treated and control HeLa cells were washed with phosphate-buffered saline and then homogenized in TRIzol (Invitrogen). Subsequently, 1 μg RNA was reverse transcribed post-extraction as previously documented [14]. Concentrations were determined by Nanodrop. We verified that equal amounts of RNA were amplified using glyceraldehyde-3-phosphate dehydrogenase (GAPDH) as a control. The expression of pro-proliferative (cyclin B, cyclin D, cyclin E, CDK2, and CDK4) and anti-proliferative (p18, p21, p27, and p53) molecules was measured. This measurement was performed using RT-PCR in OE-treated versus control groups. In this study, primer sequences were used as previously described [14].

Measurement of caspase-3 activity

The activity of caspase-3 was followed as a marker of pro-apoptotic activity. A BioVision caspase-3/CPP32 colorimetric assay kit was utilized to measure the caspase-3 activity in HeLa cells following a procedure documented previously [14].

Statistical analysis

Statistical data analysis was performed using an unpaired, two-tailed t-test, with a p-value <0.05 considered statistically significant. Each experiment was performed three times to ensure precision and limit significant errors.

## Results

Effect of OE on HeLa cell survival

**Figure 1:**
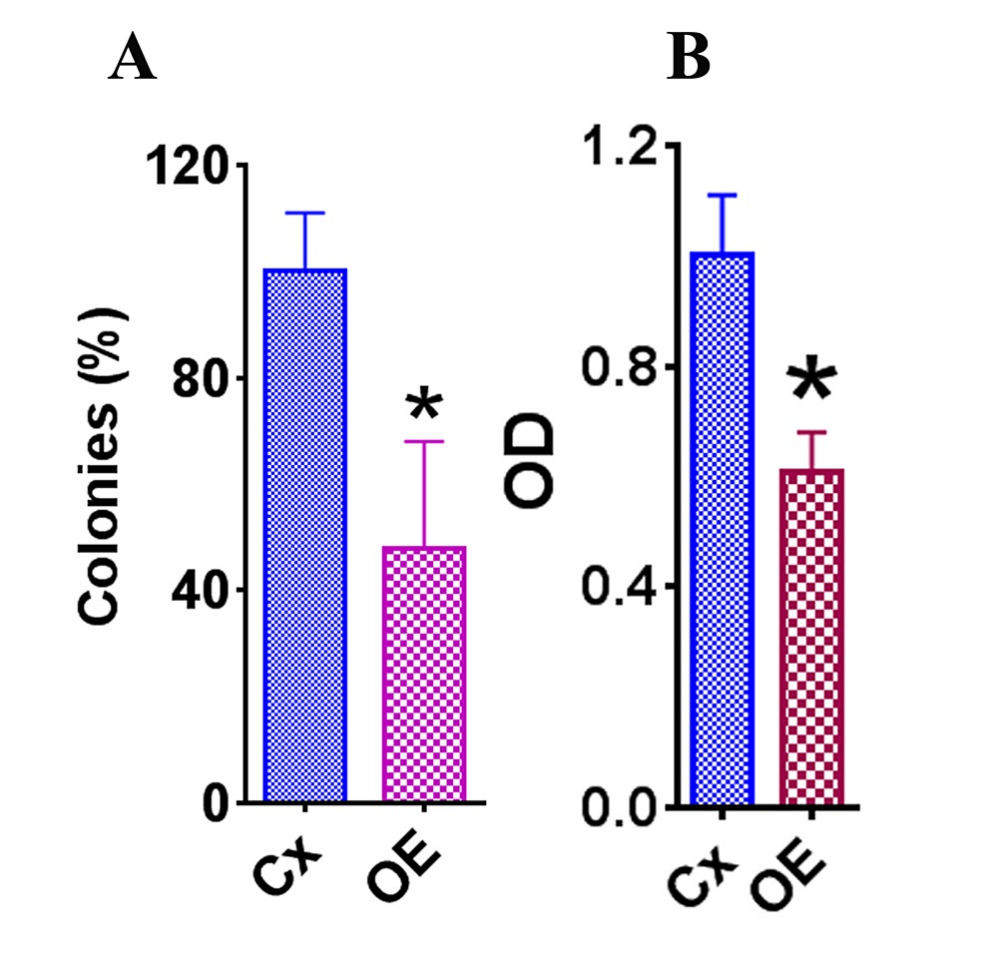
Effect of OE on survival of HeLa cells. The asterisk (*) indicates a statistically significant result in the difference in the percentage of colonies and OD in the OE groups compared to the control group (p < 0.05). (A) The clonogenic survival assay of HeLa cells treated with OE (50 μg/mL) or medium alone for 72 hours. The colony numbers were counted and expressed as a total percentage of colonies in the controls (medium alone). (B) Representative result evaluated with a cell proliferation kit between control and OE groups. These results represent two independent experiments and are expressed as the mean OD + SEM for each group. OE: olive extract; OD: optic density

HeLa cells were treated for 72 hours with OE or medium alone (control) at 70% confluence to evaluate cell survival in the presence of OE. The clonogenic survival assay showed a significant decrease in the percentage of cell colonies of HeLa cells treated with OE versus the control group (Figure 1A, p < 0.05). The optic density (OD) values that were gathered using a quick cell proliferation assay kit were significantly lower in the OE group than in the control group, which solidified the finding from the clonogenic survival assay (Figure 1B, p < 0.05). Both findings support the fact that OE negatively affects HeLa cell survival.

Effect of OE on the anti-proliferative molecule p21

**Figure 2:**
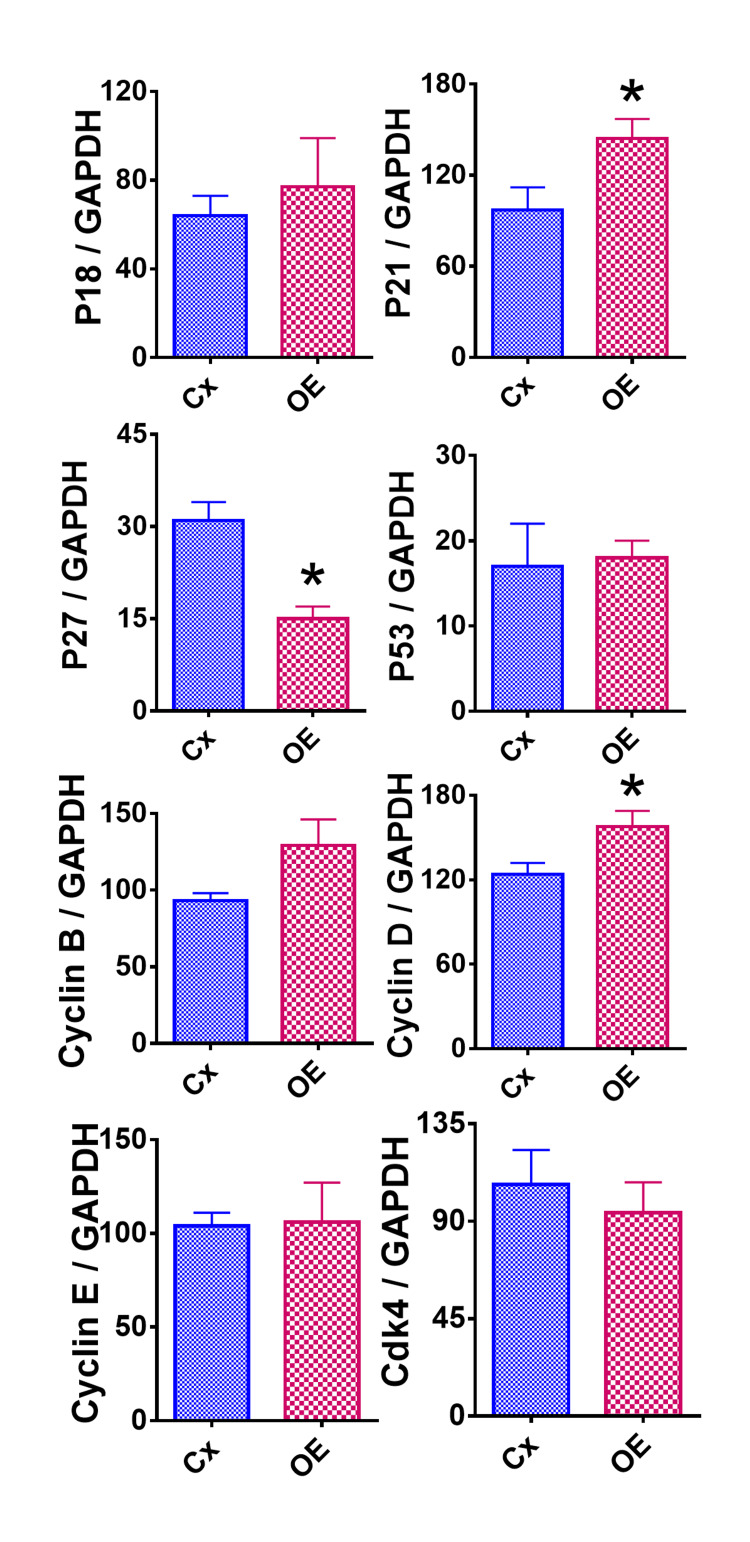
Effect of OE on mRNA expression of major pro- and anti-proliferative molecules in HeLa cells. GAPDH was used as the housekeeping gene for comparison. Experiments were completed as triplicate trials and documented on these graphs as a mean ratio of molecule densitometric units/GAPDH + SEM (×100). Each result represents two independent experiments between the control and OE groups. The asterisk (*) indicates a statistically significant difference in mRNA expression in the OE group compared to the control group (p < 0.05). OE: olive extract; GADPH: glyceraldehyde-3-phosphate dehydrogenase

**Figure 3:**
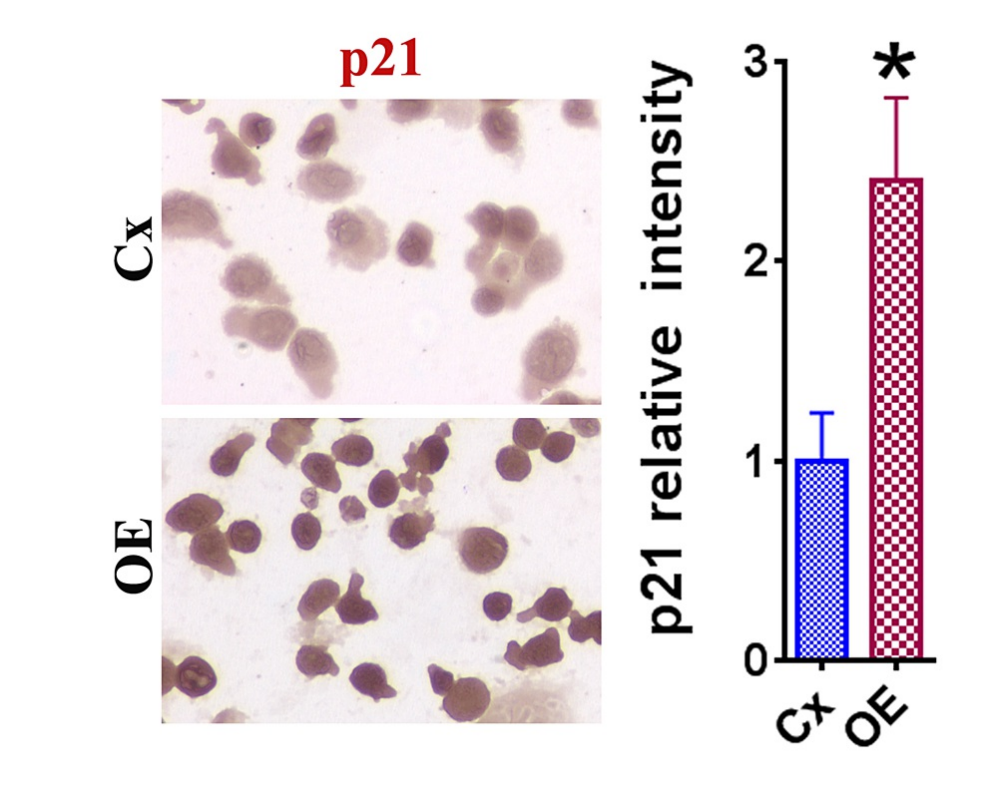
Effect of OE on the expression of p21 in HeLa cells evaluated via IHC. Representative images of IHC are displayed at the original magnification of ×400. Relative staining intensity was determined through MetaMorph image software analysis of three to five randomly selected high-power fields of three slides, relative to cells treated with medium only. The asterisk (*) indicates a statistically significant difference in staining intensity in the OE group compared to the control group (p < 0.05). OE: olive extract; IHC: immunohistochemistry

To uncover the mechanisms by which OE reduced HeLa cell survival, the expression of pro-proliferative (cyclin B, cyclin D, cyclin E, CDK2, and CDK4) and anti-proliferative (p18, p21, p27, and p53) molecules was measured. This measurement was performed using RT-PCR in OE-treated versus control groups (Figure 2). OE-treated groups showed a significant increase in the mRNA expression of p21 and cyclin D and a significant decrease in p27 (Figure 2). Together, these findings indicate that the mechanism of the net inhibitory effect of OE in HeLa cells is likely through the upregulation of p21. To further support this finding, IHC staining was performed for p21 (Figure 3). Compared to the control group, the HeLa cells treated with OE demonstrated significant increases in the relative intensity of p21 (Figure 3).

Effect of OE on apoptosis of HeLa cells

**Figure 4:**
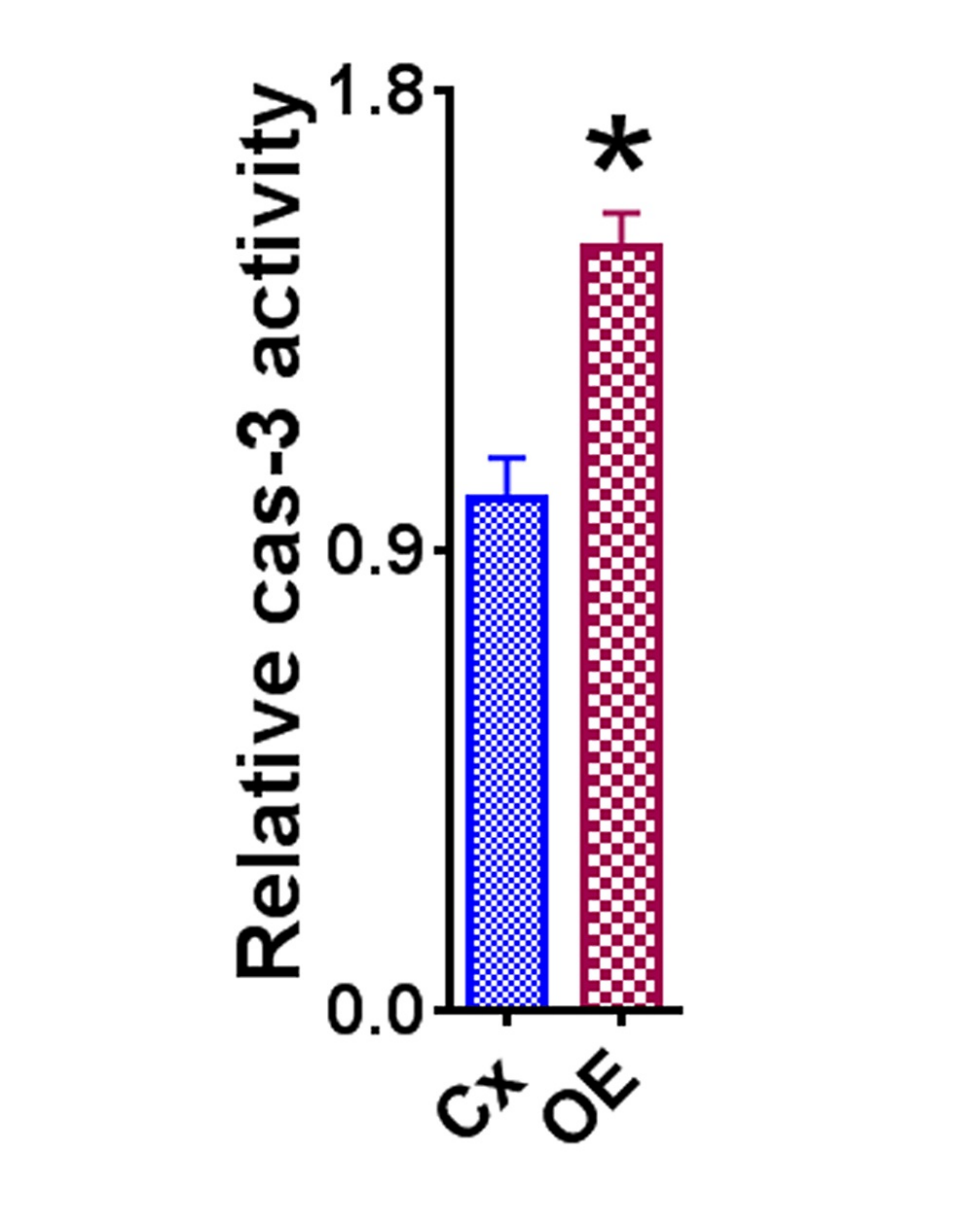
Effect of OE on apoptosis in HeLa cells. The asterisk (*) indicates a statistically significant difference in relative caspase-3 activity in the OE group compared to the control group (p < 0.05). Cellular caspase-3 activity was measured as described in the methods section and the results are expressed as mean caspase-3 activity relative to controls + SEM. Assays were completed in triplicate. OE: olive extract

To evaluate whether OE had any effects on the apoptosis of HeLa cells, in addition to its anti-proliferative effect discussed above, relative caspase-3 activity was investigated (Figure 4). Caspase-3 activity was increased in the HeLa cells treated with OE versus those in the control group. These results indicate that OE increases apoptosis of HeLa cells, contributing to the overall finding that HeLa cervical cancer cell survival is reduced in cells treated with OE.

Effect of OE on pro- and anti-apoptotic molecules

**Figure 5:**
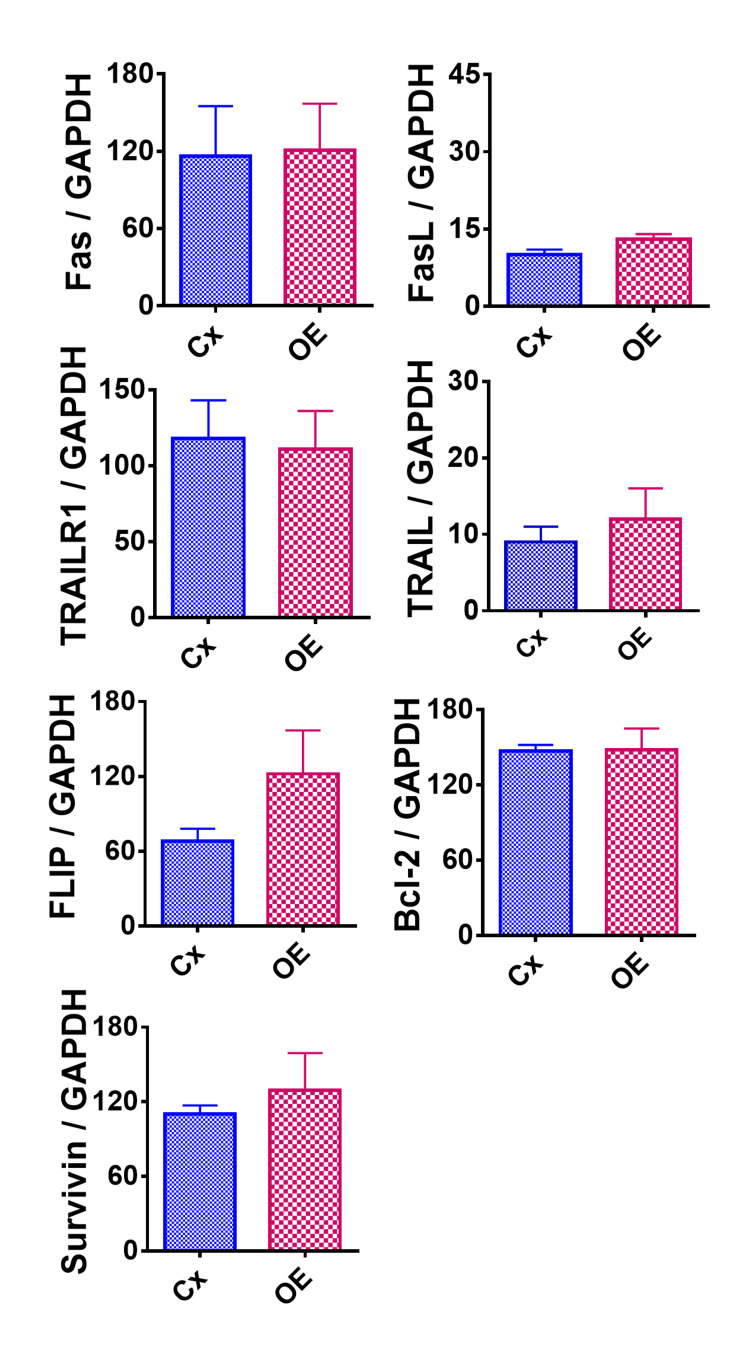
Effect of OE on mRNA expression of certain pro- and anti-apoptotic molecules in HeLa cells. GAPDH was used as the housekeeping gene for comparison. Experiments were completed as triplicate trials and documented on these graphs as a mean ratio of molecule densitometric units/GAPDH + SEM (×100). Each result represents two independent experiments between the control and OE. The asterisk (*) indicates a statistically significant difference in mRNA expression in the OE group compared to the control group (p < 0.05). OE: olive extract; GADPH: glyceraldehyde-3-phosphate dehydrogenase

To further examine the effect of OE treatment, mRNA expression levels of known important pro-apoptotic (Fas, FasL, TRAIL, and TRAIL-R1) and anti-apoptotic (FLIP, Bcl-2, and survivin) molecules were measured (Figure 5). However, there were no statistically significant changes in either pro-apoptotic or anti-apoptotic molecule expression in the OE-treated versus control group.

## Discussion

This study investigated the potential role of OE, a naturally occurring substance, on HeLa cervical cancer cell growth. It is well known that the balance, or lack thereof, between cell proliferation and cell apoptosis is tightly regulated through various molecules that govern these processes. The results of this study uncovered that OE inhibits the proliferation of HeLa cells, mainly by upregulating the anti-proliferative molecule p21. Currently, very little is known about the effects of OE on cervical cancer. To our knowledge, this is only the second study to explore the effects and mechanism of OE on HeLa cell growth in vitro [13].

The primary known cause of cervical cancer is HPV; two HPV types (16 and 18) account for about 70% of all cervical cancer, and the possible mechanism by which HPV does so has been well studied and identified [15]. One of which is that HPV contains oncoproteins E7 and E6, which override and downregulate the anti-proliferative activity of p21 [16,17]. There have been various studies in which p21 has been shown to play an important role in controlling cell cycle progression through the inhibition of various cyclin-depended kinases (CDKs) [18,19]. p21 is well-known for its vital role in cell cycle arrest. In prior studies, p21 has been shown to be upregulated by the molecule p53, which has been well-known for years to play an essential role in cell cycle regulation [18,20,21]. However, p53-independent mechanisms of p21 induction have also been defined [22,23]. One p53-independent mechanism of p21 induction is through the inhibition of maternal embryonic leucine zipper kinase (MELK) [24]. In our study, the expression of p53 in the treatment and control groups was not significantly different. However, there was a significant increase in the expression of CDK4 in the treatment group. These results suggest that a p53-dependent mechanism is likely not at play and that a p53-independent mechanism such as MELK inhibition or another mechanism of p21 upregulation was involved. Further research into the expression of MELK and other p21-interacting molecules in the OE-treated and control HeLa cells may be helpful to better understand the molecular pathways involved in this anti-proliferative effect.

Contrary to the observed effect of OE on HeLa cell growth, a significant decrease in the expression of the anti-proliferative molecule p27 was found in the treatment group. This paradoxical phenomenon has similarly been discovered in previous studies from our lab [25-28]. Another study involving radiotherapy for cervical cancer observed this same effect, where p27 was downregulated, yet cervical cancer cell growth and survival were reduced overall [29]. In this study, the authors report that broken down or dead cervical cancer cells were p27 negative, suggesting that it was the non-viable cells that were responsible for this effect. Considering this information and the results of our experiment, we infer that p27 likely either does not play a large role in cervical cancer cell growth and survival or that the results are due to p27 downregulation in deceased cells.

Furthermore, the results of the caspase-3 activity analysis indicate that HeLa cells in the OE treatment group underwent significantly more apoptosis compared to the control group. As a result, the expression of multiple pro- and anti-apoptotic molecules was determined. No statistically significant difference in the expression of these molecules was found between the OE treatment group and the control group. These results indicate a need for future research into more apoptotic regulatory molecules to help uncover the possible mechanism at play. There is some evidence that p21 is involved in the induction of apoptosis, but that a sole increase in cellular p21 is not capable of inducing apoptosis [30]. Overall, the apoptotic fate of HeLa cells may depend on a more complex interaction of these molecules or on other untested regulatory molecules.

Although limited, previous literature on this topic reveals similar findings to those uncovered by this experiment. A prior study on the effect of olive leaf extract and HeLa cells reported an upregulation of p21 paired with a downregulation of cyclin D1, leading to reduced proliferation and increased apoptosis of HeLa cells [13]. Our study showed an increase in the expression of cyclin D1 in the OE group, unlike the increase seen in this prior study. Although this difference is present, both experiments found a significant decrease in HeLa cell survival, coupled with an increase in p21 expression [13]. There is a great need for further research into this topic to uncover more about the molecular mediators in play, especially given the differences in measured cyclin D1 expression.

The findings of this experiment are promising; however, we understand that this is only an in vitro study and that there are significant limitations to making conclusions based off of this research. First, only one cervical cancer cell line (HeLa) was used. We look to evaluate the effects of OE on other cervical cancer cell lines and other cancer lines in future studies to reach a better understanding of these effects. Second, our proposed mechanism does not provide information for the pro-apoptotic effect observed and differs slightly from an earlier study on this topic, as mentioned previously. Third, our mRNA expression results do not provide context into the exact mechanism of how OE inhibits the growth of HeLa cells, given that p53 expression was not significantly altered versus the control group. This effect may be due to a p21-independent mechanism, but more research is needed to make a definitive conclusion. This does warrant evaluation of other molecules involved in p21-dependent cell cycle regulatory pathways.

## Conclusions

The leading cause of cervical cancer, HPV, causes tumor cell proliferation by decreasing the anti-proliferative effect of p21. In this study, we conclude that OE inhibited the growth of HeLa cells through the upregulation of p21. Although there are high-quality screening methods and available vaccines against HPV, cervical cancer is still the second leading cause of cancer death in middle-aged women (20-39 years old), especially those of lower socioeconomic status. Thus, any improvement in cervical cancer treatment could prevent many unfortunate and unnecessary deaths. Future research should evaluate the effects of OE, a naturally occurring substance, on other cervical cancer cell lines and explore other molecules involved in p21-dependent cell cycle regulatory pathways. Overall, this study provides a foundation for future research into the anti-proliferative effects of OE and its potential use in cancer treatment.
